# Is the Physician’s Behavior in Dyslipidemia Diagnosis in Accordance with Guidelines? Cross-Sectional Escarval Study

**DOI:** 10.1371/journal.pone.0091567

**Published:** 2014-03-13

**Authors:** Antonio Palazón-Bru, Vicente F. Gil-Guillén, Domingo Orozco-Beltrán, Vicente Pallarés-Carratalá, Francisco Valls-Roca, Carlos Sanchís-Domenech, José M. Martín-Moreno, Josep Redón, Jorge Navarro-Pérez, Antonio Fernández-Giménez, Ana M. Pérez-Navarro, José L. Trillo, Ruth Usó, Elías Ruiz

**Affiliations:** 1 Department of Clinical Medicine, Miguel Hernández University, Alicante, Spain; 2 Health Surveillance Department, Mutual Society of Castellón, Castellón, Spain; 3 Department of Medicine, Jaume I University, Castellón, Spain; 4 Health Center of Benigánim, Generalitat Valenciana, Valencia, Spain; 5 Health Center of Algemesí, Generalitat Valenciana, Valencia, Spain; 6 School of Medicine, University of Valencia, Valencia, Spain; 7 Fundación para el Fomento de la Investigación Sanitaria y Biomédica de la Comunitat Valenciana (FISABIO), Generalitat Valenciana, Valencia, Spain; 8 Conselleria de Sanitat, Generalitat Valenciana, Valencia, Spain; Northeast Ohio Medical University, United States of America

## Abstract

**Background:**

Clinical inertia has been defined as mistakes by the physician in starting or intensifying treatment when indicated. Inertia, therefore, can affect other stages in the healthcare process, like diagnosis. The diagnosis of dyslipidemia requires ≥2 high lipid values, but inappropriate behavior in the diagnosis of dyslipidemia has only previously been analyzed using just total cholesterol (TC).

**Objectives:**

To determine clinical inertia in the dyslipidemia diagnosis using both TC and high-density lipoprotein cholesterol (HDL-c) and its associated factors.

**Design:**

Cross-sectional.

**Setting:**

All health center visits in the second half of 2010 in the Valencian Community (Spain).

**Patients:**

11,386 nondyslipidemic individuals aged ≥20 years with ≥2 lipid determinations.

**Measurement Variables:**

Gender, atrial fibrillation, hypertension, diabetes, cardiovascular disease, age, and ESCARVAL training course. Lipid groups: normal (TC<5.17 mmol/L and normal HDL-c [≥1.03 mmol/L in men and ≥1.29 mmol/L in women], TC inertia (TC≥5.17 mmol/L and normal HDL-c), HDL-c inertia (TC<5.17 mmol/L and low HDL-c), and combined inertia (TC≥5.17 mmol/L and low HDL-c).

**Results:**

TC inertia: 38.0% (95% CI: 37.2–38.9%); HDL-c inertia: 17.7% (95% CI: 17.0–18.4%); and combined inertia: 9.6% (95% CI: 9.1–10.2%). The profile associated with TC inertia was: female, no cardiovascular risk factors, no cardiovascular disease, middle or advanced age; for HDL-c inertia: female, cardiovascular risk factors and cardiovascular disease; and for combined inertia: female, hypertension and middle age.

**Limitations:**

Cross-sectional study, under-reporting, no analysis of some cardiovascular risk factors or other lipid parameters.

**Conclusions:**

A more proactive attitude should be adopted, focusing on the full diagnosis of dyslipidemia in clinical practice. Special emphasis should be placed on patients with low HDL-c levels and an increased cardiovascular risk.

## Introduction

Dyslipidemia is one of the main risk factors for ischemic heart disease, which is the leading cause of death worldwide [1]–[5]. Thus, early screening for detection of dyslipidemia is a key element when attempting to prevent the complications of coronary disease. The main scientific societies recommend screening for dyslipidemia in adults [6], [7]. In Spain, the 2007 preventive activities program of the Spanish Society of Family and Community Medicine [8] only indicated in the screening process the measurement of total cholesterol (TC), whilst the 2009 program [9] recommended adding high-density lipoprotein cholesterol (HDL-c) to quantify the cardiovascular risk. Once the physician makes a diagnosis of dyslipidemia he or she should then take the appropriate action according to the relevant guidelines. This action may concern various possibilities, including dietary and hygiene measures or pharmacologic treatment [6], [8], [9].

Phillips et al [10] defined clinical inertia as mistakes by the physician in starting or intensifying treatment when indicated. Later Andrade et al defined the concept of therapeutic inertia [11]. The definition of these concepts means that inertia can affect other stages in the healthcare process, like diagnosis. Other authors have analyzed the inappropriate behavior of physicians in the diagnosis of dyslipidemia using TC (Table 1), although they did not call it clinical inertia. This behavior was assessed in several ways: lack of monitoring or diagnosis when it was required, unawareness of high blood cholesterol by the patient and not considering high blood cholesterol as a problem. All these studies involve a significant proportion of clinical inertia in the diagnosis of dyslipidemia, especially considering that it is a disease that must be controlled to reduce the incidence of coronary disease (Table 1).

**Table 1:** Main characteristics of the studies that evaluate clinical inertia in the diagnosis of dyslipidemia.

Authors	Population	N	Assessment of inertia	Inertia	Factorsassociated
Bell MMet al [12]	Adults with TC≥6.20 mmol/L andunknown dyslipidemia	93	Not diagnosing dyslipidemia	34%	TC<7.76 mmol/Land age≥70 years
Centers for DiseaseControl and Prevention(CDC) [13]	Adults with highblood cholesterol	8,112	Having high blood cholesterolwithout patient awareness	36.7%	Younger age, womenand race (blacks andMexican Americans)
Hudson JWet al [14]	Adults with TC≥5.17 mmol/L	394^†^	Not taking any action	53%	
Hyman DJet al [15]	Primary care physicians	119	Not taking any action in patientswithout cardiovascular risk factorsand TC≥5.17 mmol/L	23.7%*	
LandzbergJS et al [16]	Adults with TC≥6.85 mmol/L andunknown dyslipidemia	99	Not being treated	78%	
Levin SJet al [17]	Adults with TC≥6.20 mmol/L andunknown dyslipidemia	192	Not taking any action	80%	
Merkin SSet al [18]	Adults withhigh cholesterol	2,883	Having high blood cholesterolwithout patient awareness	47.6%	Higher educational leveland race (blacks andMexican Americans)
Naumburg EHet al [19]	Adults with TC≥6.20 mmol/L	493	Not diagnosing dyslipidemia		Minority races
Saturno HernándezPJ et al [20]	Adults with high bloodcholesterol(≥2 lipid determinations)	500	Not diagnosing dyslipidemia	88.4%	Towns>50,000 peopleand doctors withoutpostgraduate education
Steinhagen-Thiessen E et al [21]	Adults with known andunknown dyslipidemia	35,551	Not diagnosing dyslipidemiawhen the patients meetcriteria	56.7%*	Younger age, female,no DM, no hypertension,no abdominal or central obesity,no smoking, limited physicalexercise, unbalanced diet,no CVD, no otherdiseases (liver, rheumatism,arthritis or dyspnea)and higher educationallevel
StockbridgeH et al [22]	Adults with TC≥5.17 mmol/L	568	Not taking any action	17.1%*	
WhitesideC et al [23]	Adults with TC≥5.17 mmol/L	110	Not recognizing the high TCas a problem	71%	

Abbreviations: TC, total cholesterol; DM, diabetes mellitus; CVD, cardiovascular disease. *: This value was obtained through a weighted average. ^†^: The sample size is not given in the original article. We therefore obtained it from linear programming mathematical calculations based on the Simplex method.

The Valencian Community is a Mediterranean region in eastern Spain with a population of 5,004,475 inhabitants (2010 figures) [24]. The health system has universal coverage and primary care is freely accessible. There is a unique insurance number for each patient and a unique electronic health record for the whole Valencian population. In this population, TC is abnormal in approximately 50% of patients and HDL-c in one out of every four patients (NCEP criteria) [6], [25], [26]. Furthermore, patients with low HDL-c levels have a higher proportion of diabetes mellitus [26]. In Spain, the health costs of lipid-lowering medication are around €971 million per year, equivalent to 1.5% of total healthcare spending [27], [28]. Drug therapy and lifestyle modifications have a high level of cost-effectiveness in life-years gained [29], [30]. However, the noncompliance rate is around 40% for lipid-lowering drugs and 70% for lifestyle modifications [31], [32].

The ESCARVAL study (EStudio CARdiometabólico VALenciano) [33] was implemented in the Valencian Community, Spain. A cross-sectional phase of this study estimated the degree of awareness/unawareness for hypertension, dyslipidemia and diabetes, and the evolution over time of cardiovascular risk factors. Another longitudinal cardiovascular phase generated predictive scales in the general population and in patients diagnosed with hypertension, dyslipidemia and diabetes by analyzing the incidence of cardiovascular events and associated factors [33], [34].

As part of the cross-sectional ESCARVAL objectives, by means of the analysis of the electronic medical records, this present study determined the clinical inertia in the diagnosis of dyslipidemia in the population attending their health center along with the factors associated with this problem. As a new feature that adds to the work of other authors (Table 1), this study determines the clinical inertia in diagnosis taking into account the two metabolic disorders of TC and HDL-c. This resulted in determining different types of inertia in the diagnosis of dyslipidemia. The need for measures to improve the diagnosis of dyslipidemia can be seen from the results.

## Materials and Methods

### Study Population

The study population comprised all those persons who can attend their health centers in the Valencian Community. The typical profile of these persons is: mainly women, coexistence of cardiovascular risk factors, older age, and frequent visitors [35].

### Study Design and Participants

This observational, cross-sectional study analyzed a sample of nondyslipidemic individuals aged 20 years or older who had electronic medical records (Abucasis) and who attended their health center in the Valencian Community at least once between July and December 2010. We used this end time due to the delay Abucasis has in deleting deceased patients. Thus, it was certain that the information analyzed concerned patients who were alive. A patient was considered to have dyslipidemia if he or she had been diagnosed as such using ICD-9-CM codes (272.x). In addition, each patient had to have two or more lipid determinations (TC and HDL-c) in Abucasis in the second half of 2010. With these data, the physician could confirm or exclude the diagnosis of dyslipidemia, using both TC and HDL-c [8], [9]. All patients who did not meet these criteria were excluded.

### Variables and Measurements

We studied the information registered in the clinical record from when Abucasis began (May 2003) to December 2010. The patient information extracted from Abucasis included gender, a diagnosis of atrial fibrillation, hypertension or diabetes mellitus, having had cardiovascular disease (defined according to the ESH/ESC guidelines [36]), age group (20–44, 45–59, 60–74, ≥75 years) (this grouping was based on a report from the WHO) [37] and whether the physicians had done the online ESCARVAL cardiovascular skills training course, offered voluntarily and free of charge to all healthcare professionals in the Valencian Community. This online course was done in one academic year (2007–2008) and was composed of three modules: Cardiovascular Clinical Skills (Module I), Lifestyle and Dietary Hygiene Measures in the Prevention of Cardiovascular Disease (Module II), and Cardiovascular Research Skills (Module III) [33].

In addition, we extracted the average values from July to December 2010 of TC and HDL-c. According to these values, patients were grouped into categories: normal (TC <5.17 mmol/L and normal HDL-c [≥1.03 mmol/L in men and ≥1.29 mmol/L in women] [25]) and diagnostic inertia. Diagnostic inertia was defined as the patient having values of TC and/or HDL-c outside the normal range but not being diagnosed with dyslipidemia [6], [25]. We defined three modes of diagnostic inertia: 1) TC inertia: TC ≥5.17 mmol/L and normal HDL-c, 2) HDL-c inertia: TC <5.17 mmol/L and low HDL-c and 3) combined inertia: TC ≥5.17 mmol/L and low HDL-c [6], [25]. The choice of these variables was the consensus of the ESCARVAL Steering Committee. These variables are related to cardiovascular diseases. Obesity was not analyzed due to under-reporting in Abucasis.

### Sample Size

The sample size comprised 11,386 individuals who had no diagnosis of dyslipidemia in their electronic medical records. Thus, using a 95% confidence level and a maximum expected ratio (p = q = 0.5) the expected error rate in the prevalence estimation of each of the lipid categories was 0.92%.

### Statistical Analyses

Absolute and relative frequencies were used to describe the variables. Multivariable logistic regression models were performed to estimate the adjusted odds ratios (ORs) for the relationships between diagnostic inertia categories and the study variables (gender, diagnosis, age and the ESCARVAL course). Each of the diagnostic inertia categories was compared with the group of patients without inertia (normal TC and normal HDL-c). We adjusted the ORs using all the patient characteristics (gender, atrial fibrillation, hypertension, diabetes mellitus, cardiovascular disease and age group) and whether the physician had or had not done the on-line ESCARVAL course. The likelihood ratio test was carried out for the goodness-of-fit of the models. All analyses were performed at a 5% significance level and associated confidence intervals (CI) were estimated for each relevant parameter. All the analyses were performed using IBM SPSS Statistics 19.

### Missing Data

No data were missing because all the diagnoses were extracted. If the patient had no diagnosis in Abucasis, we considered that the associated variable had a negative value (no diagnosis). In addition, the ESCARVAL study has a record of all the physicians who have completed the on-line course and their patients. Finally, to formalize the clinical history of each patient it was essential to record the gender and date of birth, so there was no possibility of missing data.

### Ethical Consideration

This study was approved by the Valencian Community Public Health Ethics Committee. To comply with data protection regulations the data required for the study were requested and delivered by the principal investigator (Vicente F Gil-Guillén) to those responsible for their care. These data were supplied in unbundled, anonymized, compressed and encrypted files using a good privacy code and a cryptographic identification card of the principal investigator as an encryption key. Only the principal investigator was able to access their content. Once decrypted, computer processing was performed with the Foundation for the Promotion of Health and Biomedical Research of Valencia (FISABIO) being the sole custodian of this information. This procedure guarantees the confidentiality of the data submitted to comply with current legislation.

This population-based, non-interventional study (data from the Valencian Community) used data from medical records and informed consent was not required for included patients. The researchers informed the Valencian Community Public Health Ethics Committee about this omission (to locate the patients was impracticable). The ethics committee approved this consent procedure. This committee ensured that information access was restricted, it did not compromise the interests or welfare of any patient, it minimized the risk of injury and its use was in line with current legislation.

## Results

**Figure 1:**
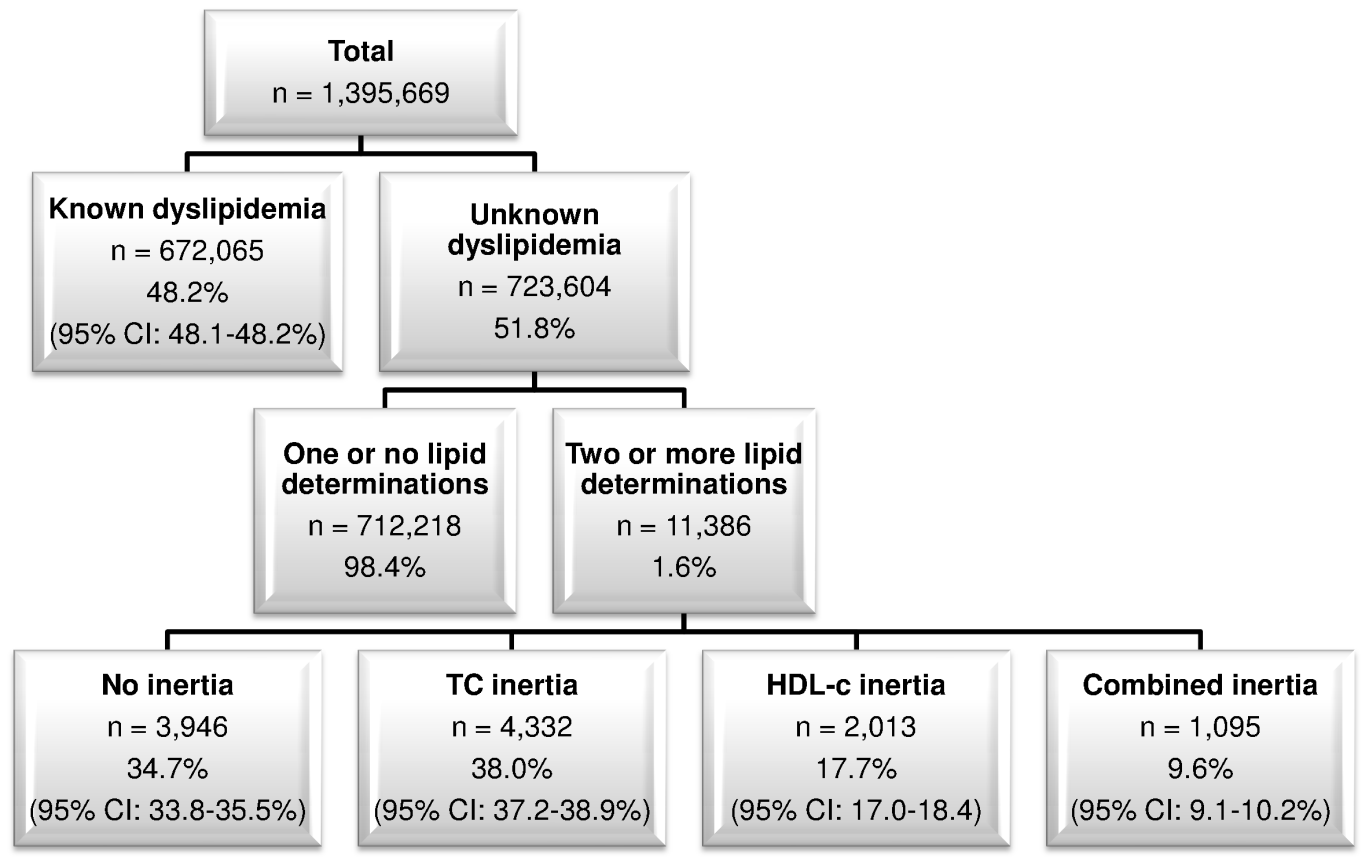
Nondyslipidemic patients at primary healthcare centers in a Spanish region. CI, confidence interval; TC, total cholesterol; HDL-c; high-density lipoprotein cholesterol.

In Figure 1 we show the number of patients in each study phase. Of a total of 1,395,669 patients who attended their health centers during the second semester of 2010, 672,065 had known dyslipidemia (48.2%, 95% CI: 48.1–48.2%) and were therefore excluded from this study. Of the remaining patients, 11,386 (1.6%) had at least two lipid profile determinations and thus entered this study. The distribution of the lipid profile groups was: 3,946 patients had no inertia (34.7%, 95% CI: 33.8–35.5%), 4,332 had TC inertia (38.0%, 95% CI: 37.2–38.9%), 2,013 had HDL-c inertia (17.7%, 95% CI: 17.0–18.4%) and 1,095 had combined inertia (9.6%, 95% CI: 9.1–10.2%).

Table 2 provides a summary of the main descriptive characteristics of the study sample. There was a higher proportion of women, a high prevalence of cardiovascular risk factors, and 7% of the participants had cardiovascular disease. The largest age group was the youngest (26.9%), and the physicians of 15.4% of the patients had done the online course. The predominance of women, and the high prevalence of cardiovascular risk factors and cardiovascular disease was present in all lipid profile categories, although there were variations in the percentages in each of the categories. In all subgroups, the percentage of physicians who did the on-line course was approximately 15%. Regarding age groups, there was wide variability in all the subgroups analyzed.

**Table 2:** Descriptive analysis of inertia groups for dyslipidemia at primary health care centers in a Spanish region.

	Total	No inertia	TC inertia	HDL-c inertia	Combined inertia
	11,386	3,946 (34.7%)	4,332 (38.0%)	2,013 (17.7%)	1,095 (9.6%)
	n(%)	n(%)	n(%)	n(%)	n(%)
**Variable**					
Gender:					
Male	4,624(40.6)	1,875(47.5)	1,588(36.7)	821(40.8)	340(31.1)
Female	6,762(59.4)	2,071(52.5)	2,744(63.3)	1,192(59.2)	755(68.9)
Atrial fibrillation:					
Yes	453(4.0)	179(4.5)	94(2.2)	144(7.2)	36(3.3)
No	10,933(96.0)	3,767(95.5)	4,238(97.8)	1,869(92.8)	1,059(96.7)
Hypertension:					
Yes	5,318(46.7)	1,825(46.2)	1,866(43.1)	1,094(54.3)	533(48.7)
No	6,068(53.3)	2,121(53.8)	2,466(56.9)	919(45.7)	562(51.3)
Diabetes mellitus:					
Yes	2,804(24.6)	1,007(25.5)	744(17.2)	771(38.3)	282(25.8)
No	8,582(75.4)	2,939(74.5)	3,588(82.8)	1,242(61.7)	813(74.2)
Cardiovascular disease:					
Yes	795(7.0)	320(8.1)	167(3.9)	245(12.2)	63(5.8)
No	10,591(93.0)	3,626(91.9)	4,165(96.1)	1,768(87.8)	1,032(94.2)
Age groups (years):					
20–44	3,058(26.9)	1,285(32.6)	955(22.0)	541(26.9)	277(25.3)
45–59	2,994(26.3)	782(19.8)	1,434(33.1)	429(21.3)	349(31.9)
60–74	2,967(26.1)	962(24.4)	1,226(28.3)	491(24.4)	288(26.3)
≥75	2,367(20.8)	917(23.2)	717(16.6)	552(27.4)	181(16.5)
On-line course by physician:					
Yes	1,757(15.4)	576(14.6)	681(15.7)	315(15.6)	185(16.9)
No	9,629(84.6)	3,370(85.4)	3,651(84.3)	1,698(84.4)	910(83.1)

Abbreviations: n(%), absolute frequency(relative frequency); TC, total cholesterol; HDL-c, high density lipoprotein cholesterol.

Table 3 summarizes the analysis of factors (gender, diagnosis, age and the ESCARVAL course) associated with each inertia group. To determine these factors we calculated the ORs, adjusted for all the patient characteristics (gender, atrial fibrillation, hypertension, diabetes mellitus, cardiovascular disease and age group) and whether the physician had or had not done the on-line ESCARVAL course. The profile of variables significantly associated (p<0.05) with TC inertia was: female (OR = 0.64, 95% CI: 0.58–0.70), no atrial fibrillation (OR = 0.59, 95% CI: 0.45–0.77), no hypertension (OR = 0.89, 95% CI: 0.80–0.99), no diabetes mellitus (OR = 0.60, 95% CI: 0.54–0.68), no cardiovascular disease (OR = 0.58, 95% CI: 0.47–0.71) and age group (in years) (20–44 → OR = 1; 45–59 → OR = 2.86, 95% CI: 2.52–3.24; 60–74 → OR = 2.44, 95% CI: 2.13–2.80; ≥75 → OR = 1.61, 95% CI: 1.38–1.89). The profile for HDL-c inertia was: female (OR = 0.68, 95% CI: 0.61–0.76), atrial fibrillation (OR = 1.37, 95% CI: 1.07–1.75), hypertension (OR = 1.22, 95% CI: 1.06–1.39), diabetes mellitus (OR = 1.82, 95% CI: 1.61–2.06) and cardiovascular disease (OR = 1.27, 95% CI: 1.04–1.54). Finally, the variables significantly associated with combined inertia were: female (OR = 0.46, 95% CI: 0.40–0.54), hypertension (OR = 1.21, 95% CI: 1.03–1.43) and age group (in years) (20–44 → OR = 1; 45–59 → OR = 2.13, 95% CI: 1.76–2.58; 60–74 → OR = 1.53, 95% CI: 1.23–1.91; ≥75 → OR = 0.97, 95% CI: 0.75–1.24). All the models were very significant (p<0.001).

**Table 3:** Analysis of factors associated with inertia groups for dyslipidemia at primary health care centers in a Spanish region.

	Adj. OR		Adj. OR		Adj. OR	
	TC inertia	p-value	HDL-c inertia	p-value	Combined inertia	p-value
	(95% CI)		(95% CI)		(95% CI)	
**Variable**						
Gender:						
Male	0.64(0.58,0.70)	<0.001	0.68(0.61,0.76)	<0.001	0.46(0.40,0.54)	<0.001
Female*						
Atrial fibrillation:						
Yes	0.59(0.45,0.77)	<0.001	1.37(1.07,1.75)	0.013	0.84 (0.57,1.24)	0.381
No*						
Hypertension:						
Yes	0.89(0.80,0.99)	0.029	1.22(1.06,1.39)	0.005	1.21 (1.03,1.43)	0.025
No*						
Diabetes mellitus:						
Yes	0.60 (0.54,0.68)	<0.001	1.82(1.61,2.06)	<0.001	1.05 (0.89,1.25)	0.534
No*						
Cardiovascular disease:						
Yes	0.58(0.47,0.71)	<0.001	1.27(1.04,1.54)	0.017	0.80(0.59,1.07)	0.135
No*						
Age groups (years):						
20–44*		<0.001		0.090		<0.001
45–59	2.86(2.52,3.24)		1.14 (0.97,1.35)		2.13(1.76,2.58)	
60–74	2.44(2.13,2.80)		0.92 (0.77,1.11)		1.53 (1.23,1.91)	
≥75	1.61 (1.38,1.89)		0.98 (0.82,1.19)		0.97(0.75,1.24)	
On-line course by physician:						
Yes	1.12(0.99,1.27)	0.078	1.04(0.89,1.21)	0.654	1.18 (0.98,1.42)	0.078
No*						

Abbreviations: TC, total cholesterol; HDL-c, high density lipoprotein cholesterol; Adj. OR, Adjusted Odds Ratio; CI, Confidence Interval.

Goodness-of-fit of the inertia models: TC: *X^2^* = 552.7, p<0.001; HDL-c: *X* ^2^ = 182.9, p<0.001; Combined: *X* ^2^ = 205.7, p<0.001.

OR adjusted for gender, atrial fibrillation, hypertension, diabetes mellitus, cardiovascular disease, age groups and the on-line course.

^*^: Reference.

## Discussion

In our study almost four out of every ten patients had diagnostic inertia of their TC, one in six had diagnostic inertia of their HDL-c, and one in ten had combined inertia. A search of the literature showed studies evaluating physician behavior in the diagnosis of dyslipidemia (Table 1). These papers involve populations that differ greatly from our study population, in addition to having different designs, and where this problem does not have this particular name (inertia) (Table 1). The rate of inertia found in these studies ranged from 17.1–88.4%. All these papers considered just TC, so we can only compare them with our results for TC inertia alone. Our magnitude of TC inertia was below the mean and median weighted by number of patients (52.7 mean, median 56.7). This indicates that although in our population TC inertia is a prevalent problem, it is still lower than in other countries.

Factors associated with TC inertia in our study were female; not having atrial fibrillation, hypertension, diabetes mellitus or cardiovascular disease; and middle and advanced age. The other authors (Table 1) reported similar findings, as well as detecting greater inertia among lower TC levels, certain racial groups, and in association with social factors, other diseases (liver, dyspnea, rheumatism and arthritis), unbalanced diet, limited physical exercise and postgraduate medical training. Differences were also present in the studies consulted regarding age (Table 1).

Considering that in Spain HDL-c is used to diagnose dyslipidemia and that we have found no studies evaluating behavior in the diagnosis of dyslipidemia using HDL-c, we decided to conduct an analysis in this lipid parameter to quantify the inertia and its associated factors. The results obtained are of concern as almost one in every four patients had an abnormal HDL-c level that was not recognized by the physician (HDL-c inertia 17.7%, combined inertia 9.6%). But, even more worryingly, the profile of the factors associated with HDL-c inertia concerned young people, women, and with cardiovascular risk factors and cardiovascular disease. Furthermore, when abnormal TC and HDL-c were combined, cardiovascular disease lost its statistical significance. Regarding age, combined inertia was more common in middle age than in younger people, like HDL-c inertia.

When we started the study, we expected to find a lower magnitude of inertia and that patients with inertia would have a lower cardiovascular risk. However, the results surprised us greatly, especially the high prevalence of inertia in all its forms; and this considering that many of these patients are diagnosed with other cardiovascular risk factors and they should have their lipid profile monitored to prevent ischemic heart disease. In addition, we are concerned that physicians did not assess HDL-c in patients with a very high cardiovascular risk or in those who had a cardiovascular disease. A possible reason that we consider important and which may justify the conservative attitude of clinicians in patients with a low HDL-c is the clinical difficulty to raise these levels, as currently available drugs are not very effective and lifestyle modifications experience minimum adherence by the patient [6], [32].

Our results suggest that healthcare policies should be active in the fight against coronary heart disease through the detection and treatment of its risk factors, like dyslipidemia. However, the Valencian Community is experiencing an epidemic of obesity resulting in an increased prevalence of cardiovascular risk factors [26]. Our findings indicate the need to integrate these healthcare policies in the health centers, identifying the dyslipidemic patient early and controlling the situation through drug treatment and lifestyle changes to reduce the incidence of ischemic heart disease in the population.

### Search Equation

The papers used for comparative purposes were found in MEDLINE using the following keywords: cholesterol, hypercholesterolemia, cholesterol HDL, cholesterol LDL, hyperlipidemias, physician, provider, doctor, nurse, professional, routine, style, manner, action, intervention, practice, experience, conduct, adherence, guidelines, guide, behavior, behaviour, knowledge, ignorance and awareness. The filters used were: abstract available, humans and adult (19+ years).

### Study Limitations and Strengths

The source of information corresponds to a unique electronic record that integrates all the healthcare information from the health centers. In addition, this paper comprehensively addresses the novel problem of clinical inertia in dyslipidemia diagnosis. Furthermore, the sample size is large, minimizing random error when drawing conclusions from the results obtained in the population visiting health centers. In addition, the fact that all the health centers in the Valencian Community participated in this study and we quantified the problem of inertia in the decisions of all the members of the primary care teams provides our conclusions with external validity. This means that our results can be generalized to populations with a health system similar to ours, i.e. universal, public, freely available, and without charge to patients. It would therefore be interesting to conduct similar studies in other countries with different health policies through projects that have large numbers of patients and healthcare professionals.

The limitations of this study are defined by the design. Since this was a cross-sectional study, it is not possible to establish a temporal sequence between the factors and the dependent variable (inertia), although the status of undiagnosed dyslipidemic patients can be assessed and their needs determined. These elements are key in combating unawareness of this problem and prioritizing healthcare planning. The most important bias in this study may be that which is accepted in this type of study, i.e., selection bias. This bias is related to the fact that it is the most motivated patients who go to the health centers. Logically, this cannot be changed as each person has a different degree of healthcare motivation. However, it does not affect the aims of this study because we are quantifying the phenomenon of inertia or a conservative or tolerant attitude by primary care teams when diagnosing dyslipidemia. Another weakness of this study concerns under-reporting in the medical history by healthcare professionals. To minimize this bias, all the physicians were given the opportunity to participate, voluntarily and free of charge, in the on-line ESCARVAL course, which provides training on cardiovascular disease and its risk factors. Furthermore, as computerized drug prescription is mandatory, knowing which prescription drugs each patient had been prescribed enabled us to determine each patient’s disorders, thus minimizing the under-reporting that is always assumed in these types of studies. Finally, blood test results (specifically lipids) are automatically registered in Abucasis, through the electronic laboratory. A further limitation is not having analyzed other cardiovascular risk factors, like a family history of cardiovascular disease, diet, lifestyle, and obesity [6]. This was due to the lack of these data on the medical records completed by the healthcare professionals, although these variables would be more beneficial for the longitudinal ESCARVAL study [33], [34]. Finally, we did not use another parameter for the detection of dyslipidemia (low-density lipoprotein cholesterol or triglycerides) as in Spain TC and HDL-c are the only parameters recommended for this process [8], [9].

### Conclusion

We think that this study is ideal to quantify the clinical inertia in the diagnosis of dyslipidemia through computerized systems in the community. The main point arising from this paper is that we have to adopt a more proactive attitude towards dyslipidemia. This attitude should focus on the full diagnosis of dyslipidemia in clinical practice when a patient meets the criteria, making the diagnosis as soon as possible. Special emphasis must also be given to patients with low HDL-c levels and an increased cardiovascular risk.

Finally, it would be interesting to integrate alarm systems in the computerized health records system aimed at reducing this problem, alerting the clinician when a patient has the diagnostic criteria so that suitable treatment can begin immediately. This could reduce the inertia and thus the incidence of ischemic heart disease.
